# Dr. Margaret E. Hennessey

**Published:** 1915-10

**Authors:** 


**Dr. Margaret E. Hennessey,** Hahnemann of Chicago 1888, died of cancer of the uterus at La Salle, Ill., April 23, aged 58. She was a practitioner of Utica.

